# Model SNP development for complex genomes based on hexaploid oat using high-throughput 454 sequencing technology

**DOI:** 10.1186/1471-2164-12-77

**Published:** 2011-01-27

**Authors:** Rebekah E Oliver, Gerard R Lazo, Joseph D Lutz, Marc J Rubenfield, Nicholas A Tinker, Joseph M Anderson, Nicole H Wisniewski Morehead, Dinesh Adhikary, Eric N Jellen, P Jeffrey Maughan, Gina L Brown Guedira, Shiaoman Chao, Aaron D Beattie, Martin L Carson, Howard W Rines, Donald E Obert, J Michael Bonman, Eric W Jackson

**Affiliations:** 1 USDA-ARS, Small Grains and Potato Germplasm Research Unit, Aberdeen, ID, USA; 2 USDA-ARS, Western Regional Research Center, Albany, CA, USA; 3 General Mills Agriculture Research, LeSueur, MN, USA; 4 Beckman Coulter Genomics, Beverly, MA, USA; 5 Agriculture and Agri-Food Canada, Ottawa, ON, Canada; 6 USDA-ARS, Dept. Agronomy, Purdue University, West Lafayette, IN, USA; 7 Dept. Plant and Wildlife Sciences, Brigham Young University, Provo, UT, USA; 8 USDA-ARS, Plant Science Research, Raleigh, NC, USA; 9 USDA-ARS, Cereal Crops Research, Fargo, ND, USA; 10 Crop Development Centre, University of Saskatchewan, Saskatoon, SK Canada; 11 USDA-ARS, Cereal Disease Laboratory, St. Paul, MN, USA; 12 Dept. Agronomy and Plant Genetics, University of MN, St. Paul, MN, USA; 13 Current Address: Knome, Inc., Cambridge, MA, USA

## Abstract

**Background:**

Genetic markers are pivotal to modern genomics research; however, discovery and genotyping of molecular markers in oat has been hindered by the size and complexity of the genome, and by a scarcity of sequence data. The purpose of this study was to generate oat expressed sequence tag (EST) information, develop a bioinformatics pipeline for SNP discovery, and establish a method for rapid, cost-effective, and straightforward genotyping of SNP markers in complex polyploid genomes such as oat.

**Results:**

Based on cDNA libraries of four cultivated oat genotypes, approximately 127,000 contigs were assembled from approximately one million Roche 454 sequence reads. Contigs were filtered through a novel bioinformatics pipeline to eliminate ambiguous polymorphism caused by subgenome homology, and 96 *in silico *SNPs were selected from 9,448 candidate loci for validation using high-resolution melting (HRM) analysis. Of these, 52 (54%) were polymorphic between parents of the Ogle1040 × TAM O-301 (OT) mapping population, with 48 segregating as single Mendelian loci, and 44 being placed on the existing OT linkage map. Ogle and TAM amplicons from 12 primers were sequenced for SNP validation, revealing complex polymorphism in seven amplicons but general sequence conservation within SNP loci. Whole-amplicon interrogation with HRM revealed insertions, deletions, and heterozygotes in secondary oat germplasm pools, generating multiple alleles at some primer targets. To validate marker utility, 36 SNP assays were used to evaluate the genetic diversity of 34 diverse oat genotypes. Dendrogram clusters corresponded generally to known genome composition and genetic ancestry.

**Conclusions:**

The high-throughput SNP discovery pipeline presented here is a rapid and effective method for identification of polymorphic SNP alleles in the oat genome. The current-generation HRM system is a simple and highly-informative platform for SNP genotyping. These techniques provide a model for SNP discovery and genotyping in other species with complex and poorly-characterized genomes.

## Background

Genetic markers accurately distributed throughout a genome are pivotal to the success of association mapping, marker-assisted breeding (MAB), map-based cloning, and studies relating to genome structure and function. Single nucleotide polymorphisms (SNPs) are the most abundant type of DNA variation currently used as genetic markers [1]. SNP assays directly interrogate the sequence variation, reducing genotyping errors compared to assays based on size discrimination or hybridization. SNP assays are also amenable to high-throughput technologies, making them an excellent tool for use in modern genomics research.

Advances in sequencing technology have enhanced genome-wide SNP discovery, and SNP platforms have been developed for a number of diploid species. These resources have enabled comprehensive genetic analyses in barley [2,3], rice [4], maize [5], and soybean [6,7], including studies on diversity and population structure, comparative genomics, and QTL identification [3,5,8-11].

As with SNP discovery methods, SNP genotyping technologies have proliferated in recent years, with available platforms utilizing mass spectroscopy [12], direct sequencing, fluorescence detection, and microchip hybridization [13]. Each technology has advantages and limitations. For example, the widely utilized TaqMan^® ^assay has a high sample success rate, with excellent repeatability and cluster separation [14] but requires fluorescence-labeled probes, making the assay cost prohibitive for large numbers of assays. The Illumina GoldenGate assay [15,16] is also widely used, but limited to allele differentiation, and is cost-effective only for a large number of SNPs run in a single parallel assay. A practical and more flexible alternative may be available using amplicon melting analysis in conjunction with real-time PCR [17]. This method uses unlabeled primers and interrogates the entire amplicon, providing an efficient SNP genotyping system in terms of reagent cost, throughput, and data production.

Although direct detection of sequence variation is robust and accurate, the requirement of sequence information for SNP discovery has been an obstacle in complex uncharacterized genomes with limited funding, including cultivated oat (*Avena sativa *L.). Current-generation Roche 454 pyrosequencing now allows cost-effective *de novo *sequencing and assembly with excellent depth and coverage, low error rates, and rapid output [18,19]. Novaes et al. [20] sequenced transcriptomes from six tissue types of seven *Eucalyptus grandis *(2n = 2x = 22) families using 454 technology. The sequencing effort generated 148 Mbp of expressed sequence that assembled into 2,392 contigs. Comparison of individual reads with a consensus assembly detected 23,742 putative SNPs. These results demonstrate that current-generation transcriptome sequencing can overcome obstacles in crops with sparse sequencing resources; however, additional work is needed to apply these high-throughput SNP-discovery approaches to polyploid plant genomes.

Utilization of next-generation sequencing for SNP discovery requires significant data management and analysis of sequence information. A system for handling data complexity has been developed for pine [21], and could serve as a model for SNP mining in other species. The method runs on a Unix/Linux platform written in Perl, using Phred for base calling, and Phrap and ProbconsRNA for sequence alignment. Individual alignments are converted to FASTA files and aligned to an overall consensus. SNPs are called using a customized WEKA classifier package and validated manually with Phrap. The hybrid program increased the speed and accuracy of SNP calls.

Effective SNP discovery in complex genomes would require additional analysis to consider duplicate loci and to identify and eliminate pseudo-SNPs produced by misassembly of paralogous and homoeologous sequences inherent to polyploid genomes. For example, cultivated wheat, including common wheat (*Triticum aestivum*, 2n = 6x = 42) and durum wheat (*T. turgidum*, 2n = 4x = 28), has been the subject of intense genetic investigation, but SNP discovery and assay development have not kept pace with other species of similar importance (e.g., rice). A recent report by Akhunov et al. [22] demonstrated the feasibility of using the Illumina GoldenGate assay for SNP development in both tetraploid and hexaploid wheat genomes, but also underlined the complications presented by multiple targets in polyploid genomes. Although gene duplication is frequent even in diploid genomes [23,24], redundant loci are far more prevalent within polyploid genomes, complicating the discrimination of haplotypes and allele ratios [25,26]. Complications may also arise if polymorphisms are discovered through transcriptome analysis, since the transcriptome only represents copies of expressed alleles. For example, Adams et al. [27] showed that homoeologous genes in cotton produced various levels of gene silencing, with expression biased in different genes and tissues. If not accounted for, these homoeologous gene sets could distort and prevent marker development in regions with subgenome homologies.

Genome assembly will be especially complicated in cultivated oat, where partial homoeology, numerous chromosomal rearrangements, and *cis- *and *trans- *sequence duplication exist within the genome. Oat research is also handicapped by a lack of DNA sequence information. GenBank holds only a few thousand ESTs, most of which are from a limited number of genotypes and tissues [28].

Several attempts have been made to develop good marker resources in oat. Microsatellite technology has produced several hundred oat markers [29-34]; however, the number of robust markers available for routine laboratory use is limited [35]. Recently, a high-throughput marker array based on DArT technology was developed for cultivated oat [36]. These markers have quickly become the marker of choice for the oat community; however, efficient and repeatable use of this technology has thus far been limited to a single service provider, and the marker produces a dominant polymorphism which does not allow discrimination of both alleles at a locus. The lack of numerous, easily assayed, and co-dominant markers remains a major barrier in oat genetics research. Whole-genome SNP discovery is, therefore, a high priority to advance genetics research in this complex genome.

Here, we report a method for high-throughput SNP discovery in cultivated oat, a species with a large, complex, and uncharacterized genome. Specific objectives were 1) to develop genotype-specific transcriptome libraries from four cultivars, 2) to mine SNPs by comparative alignment of transcriptome data, 3) to validate SNPs with new-generation melt curve analysis, 4) to determine putative SNP positions on the current OT linkage map, and 5) to utilize the SNP loci to study genetic diversity in a panel of oat germplasm.

## Results

### RNA extraction

Mean quantities of RNA included 66 μg from shoot tissue, 39 μg from roots, 84 μg from pistillate structures, and 122 μg from mature embryos (Table 1). Individual tissue samples were pooled within a genotype, yielding, on average, 311 μg RNA from each of the four oat genotypes.

**Table 1:** RNA used for cDNA library construction

Genotype	RNA (μg)				Total RNA (μg)	Ratio
	**Shoot**	**Root**	**Pistillate structures**	**Mature embryo**		

Ogle1040	68.00	50.77	26.62	173.14	318.53	1:0.75:0.39:2.55
TAM O-301	73.28	19.33	93.28	43.31	229.20	1:0.26:1.27:0.59
Gem	52.10	66.70	62.28	164.17	345.25	1:1.28:1.20:3.15
HiFi	70.80	20.67	155.02	105.87	352.36	1:0.29:2.19:1.50
Mean	66.05	39.37	84.30	121.62	311.34	1:0.60:1.28:1.84
Total	264.18	157.47	337.20	486.49	1245.34	

### Roche 454 GS-FLX sequencing and contig assembly

Read depth and coverage were greatest for TAM O-301, followed by Ogle1040, HiFi, and Gem. Sequencing runs generated between 180,447 and 381,397 reads per genotype (Table 2). Approximately 80% of reads assembled into contigs, with a mean of 31,777 contigs per genotype and total contig length ranging from 14.2 to 24.7 Mb. Individual contig size ranged from 40 to 4416 bp, with an overall mean of 577 bp and mean SD of 220. Average transcript coverage for the four genotypes ranged from 3.1× to 4.1×, with a mean of 3.7×.

**Table 2:** 454 Sequencing data from cDNA libraries of four oat genotypes

Genotype	Total reads	Assembled reads	# contigs	Total contig length (Mb)	Mean contig length (bp)	length SD (bp)	Minimum contig size (bp)	Maximum contig size (bp)	Average coverage (x)
Ogle1040	283,478	231,057	32,245	18.09	561	205	41	2217	4.1
TAM O-301	381,397	307,751	42,147	24.70	586	217	40	4303	4.0
Gem	180,447	137,743	23,681	14.20	598	238	43	4229	3.1
HiFi	235,589	188,015	29,036	16.40	564	220	42	4416	3.6
Total	1,080,911	864,566	127,109						

**Figure 1:**
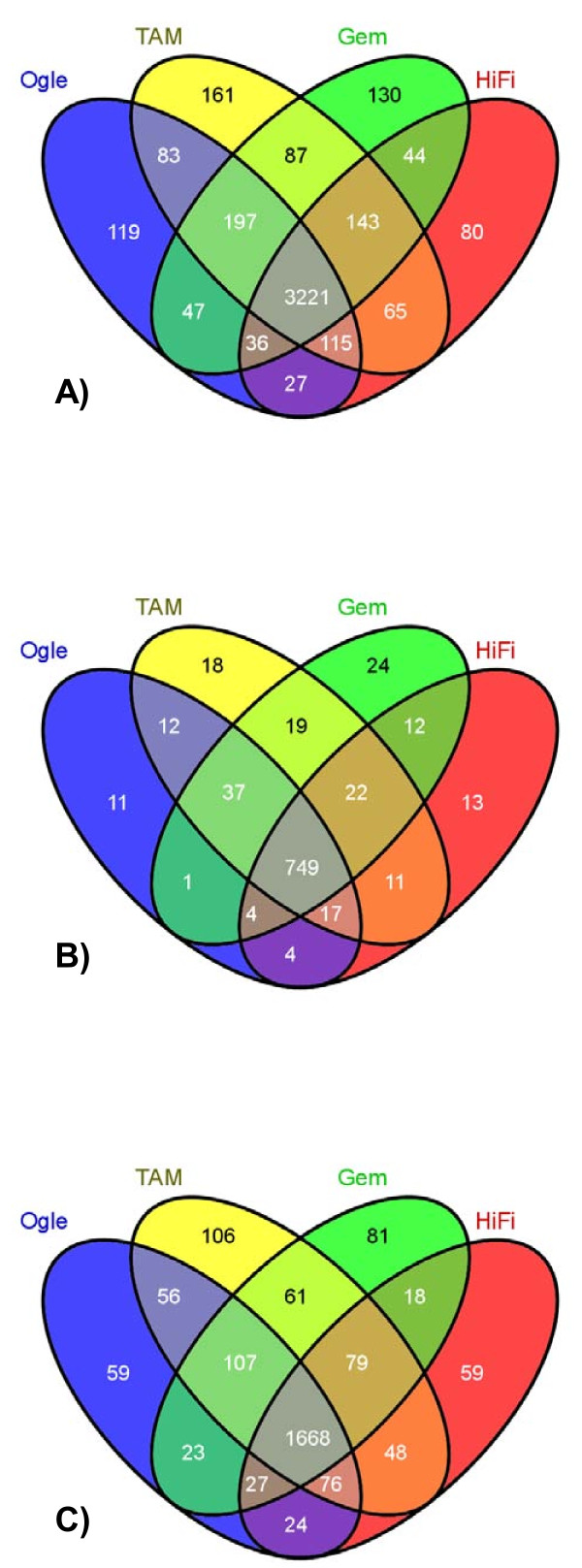
**Gene Ontology (GO) comparisons**. Comparison of biological (A), cellular(B), and molecular (C) GO terms associated with germplasm accessions Ogle1040, TAM O-301, Gem, and HiFi. Term overlaps are shown with a four-way Venn diagram constructed using VENNY. The shared areas for all germplasm were 83% (3221), 88% (749), and 80% (1668) for the different GO terms assigned to the biological, cellular, and molecular GO term classifications, respectively. The numbers of GO terms matched to assembled sequences were 12260 (Ogle), 16040 (TAM), 9605 (Gem), and 11156 (HiFi).

Of the contigs formed from the four germplasm accessions, matches < e-10 to the UniProt database by BLASTX numbered 9814, 11374, 12525, and 16333 for cvs. Gem, HiFi, Ogle, and TAM, respectively (approximately 40% match across cultivars). Contigs were categorized by GO molecular, biological, and cellular terms and the gene classes in common to all cultivars ranged from 80 to 88% (Figure 1).

### *in silico *SNP detection and assay development

Contig assemblies from TAM sequence reads were most numerous (42,147 contigs), and were therefore used as the reference assembly (RA) for *in silico *SNP detection. Comparison of genotype-specific reads with the RA indicated that 61% (25,908), 40% (17,101), and 50% (21,089) of the RA were covered by contigs from Ogle, Gem, and HiFi, respectively. The SNP screening pipeline was based on re-assembly of reads from each genotype against the RA using Roche GS Reference Mapper software, identification of all SNP-containing contigs, and elimination of those not meeting a set of criteria. The first step was the elimination of polymorphisms identified within the TAM re-assembly. Although these could be sequencing errors, they would interfere with clear identification of other SNPs. Of the 42,147 reference contigs, 2,179 (5%) were eliminated due to insertion-deletion (indel) polymorphism and 10,514 (25%) contigs were removed because of ambiguous base calls (N). When comparing the remaining 51,405 reads to the RA, 7,041 (14%) were removed based on indels, and 6,874 (13%) were removed based on insufficient read depth (≤ 4). The greatest attrition in candidate SNPs, however, was based on SNP conservation between reads of a single germplasm (selecting 100% consistency within genotypes). In this case, 28,042 (55%) *in silico *SNPs were rejected. This conservative *in silico *SNP selection method produced 9,448 candidate SNP loci (18%) for assay development, of which 8,408 (16%) interrogated SNP variation between a single genotype and the RA, and 953 (1.8%) and 87 (0.2%) interrogated variation between the RA and two or three genotypes, respectively.

In this study, the class containing SNP variations between all three genotypes and the RA (87) was chosen to mine SNPs for assay development. This subset was chosen to maximize mapping efficency in the OT population and to identify a small representative cross section of loci. Of these, 16 contigs were eliminated because they contained multiple SNP targets within a 100 bp region, which could be problematic for successful assay development. Sequences from the remaining 71 contigs were imported into the BatchPrimer3 v1.0 software [37]. A total of 301 candidate SNP assays were designed, of which 96 SNPS representing 71 unique RA contigs were selected for validation.

### SNP assay validation

**Figure 2:**
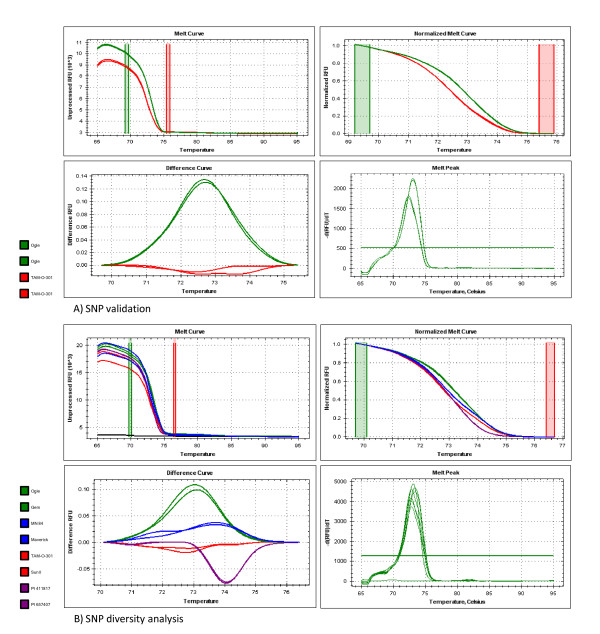
**High-resolution melt analysis**. Examples of SNP validation (A) and SNP diversity analysis (B) using primer oat EST SNP c22314_1. In example (B), the melting curves of primary alleles are shown red and green, those of heterozygous genotypes in blue, and those of genotypes found to contain a deletion within the amplicons in purple.

The 96 assays selected to interrogate previously described SNP loci were validated in Ogle and TAM and the 136 recombinant inbred lines of the Ogle × TAM (OT) population [38]. Sixty-seven primers (69.8%) generated a robust reaction between the parents, with 52 primers producing clear reactions across the OT progeny. Of these, four revealed a high proportion (approximately 50%) of heterozygous genotype calls. These were not considered markers because the heterozygous class could indicate a mixture of alleles at two different loci. The remaining 48 primers yielded bi-allelic reactions with minimal segregation distortion (Figure 2) and were added to the OT linkage map. Forty-four markers mapped to 17 linkage groups and one fragment group, with four markers remaining unlinked (Table 3). In general, SNP map positions correlated with previous marker density, with more SNP markers mapping to larger or more densely-mapped linkage groups.

**Table 3:** SNP allele, map position, and segregation ratio of markers polymorphic in the Ogle1040 × TAM O-301 (OT) mapping population

SNP	Allele		OT LG^a^	SR^b^
			
	Ogle1040	TAM O-301		
C51_1	T	C	OT_4	0.90
C51_2	C	T	OT_4	0.81
C104_1	G	A	OT_30	0.49
C250_1	A	G	OT_20_29	2.00
c318_1	G	C	OT_34	1.61
c540_1	A	G	Frag_14	1.08
c841_2	T	G	OT_32_33	0.71
c841_3	T	G	OT_32_33	0.68
c876_1a	T	G	OT_16	0.89
c876_1b	A	T	OT_10	1.32
c1196_1	A	T	OT_6	1.30
c1361_1	C	A	OT_32_33	0.91
c1579_1	G	C	OT_20_29	1.08
c2043_1	C	G	OT_31	0.91
c2106_2	-	-	OT_32_33	0.96
c2391_1	C	T	OT_32_33	0.68
c2391_2	C	T	OT_30	0.82
c2391_4	C	G	OT_34	1.01
c2391_5	G	T	OT_34	1.09
c2539_1	-	-	Unlinked	1.60
c2680_1	T	C	OT_24	0.94
c3212_1	A	G	OT_24	1.48
c3768_1	G	A	OT_24	1.03
c4096_1	-	-	OT_13	0.86
c5153_1	G	A	OT_27	0.79
c5252_1	G	A	OT_11	0.94
c5469_1	A	G	OT_1	1.05
c7461_1	G	A	OT_2	1.08
c10486_1	T	G	Unlinked	0.92
c11164_1	A	G	OT_11	1.16
c11164_2	C	T	OT_6	1.21
c12344_1	C	G	Unlinked	0.79
c12516_1	T	A	OT_27	0.82
c12516_2	G	A	OT_34	1.08
c14852_2	C	A	OT_2	0.78
c15098_1	C	T	OT_34	1.00
c16908_1	C	A	OT_10	0.73
c22314_1	G	T	OT_11	0.94
c23257_1	A	C	OT_13	0.96
lrc14030_1	T	A	OT_27	0.79
lrc16053_1	A	G	OT_8	1.13
lrc16053_2	C	T	OT_32_33	0.67
lrc16053_3	T	C	OT_32_33	0.89
lrc27472_2	A	C	OT_32_33	0.68
lrc34490_1	C	A	OT_15	0.92
lrc38531_1	C	A	OT_34	0.94
lrc38531_2	-	-	OT_8	0.75
lrc40347_1	G	C	Unlinked	0.81

^a ^Linkage group assignment is based on appending new SSR loci to the existing OT linkage map [Ref. [35]] using Map Manager QTX.

^b ^SR = Segregation ratio, calculated as the quotient of O/T allele frequencies within the population.

### Diversity analysis

The 36 SNP markers used for diversity analysis produced 140 alleles, an average of 3.89 alleles per marker, suggesting a high frequency of allelic variation. This variation was categorized as 73 alternate SNP alleles, and 67 non-SNP alleles, including 51 insertions or deletions, 6 probable heterozygotes, and 10 null alleles. Although all markers except RA c14852_2 were multi-allelic, supplementary alleles were confined primarily to non-*sativa *species (Additional file 1). Frequent allele variants were also observed in cultivars Kangaroo and CI4706-2, genotypes that represent unique origins, and likely unique genetic backgrounds, within this study (Additional files 1, 2). Indels and heterozygous genotypes were less common in other accessions.

**Figure 3:**
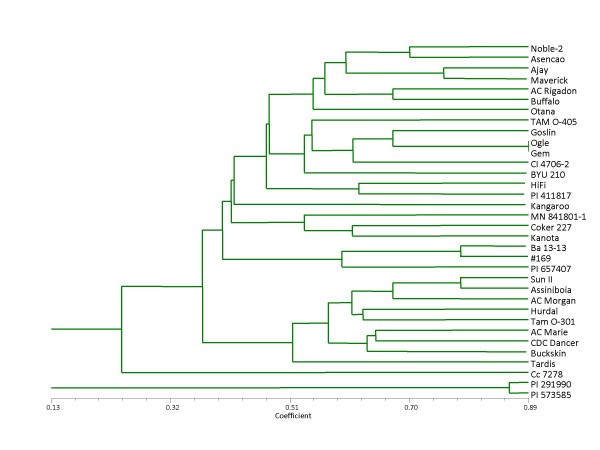
**Cluster analysis**. Diversity analysis of oat genotypes was based on the UPGMA method and EST-derived oat SNP markers. PI 291990 and PI 573585 are A-genome diploids (*A. strigosa*); Cc 7278 is a C-genome diploid (*A. eriantha*); #169 and Ba 13-13 are CD-genome tetraploids (*A. magna*); and BYU 210 is a AC-genome tetraploid (*A. insularis*). Remaining genotypes are hexaploid.

Cluster analysis separated genotypes primarily according to genome constitution, geographic origin, and genetic ancestry (Figure 3, Table 4). Diploid species were genetically distinct: *A. eriantha *(genome C_p_C_p_) diverged at the first node and the *A. strigosa *accessions (genome A_s_A_s_) were disjoined from the cladogram. The remaining 33 genotypes separated into two major clades, with much of the cluster separation correlating with ancestry. For example, cultivars Ajay and Maverick, which share a clade, are derived from the same breeding program, and pedigrees of both genotypes include Otana, represented on a different branch within the clade. Terminal clades of cultivars Ogle and Gem, and Sun II and Assiniboia, are similarly explained: in both pairs, one genotype is prominent in the pedigree of the other (Additional file 2, pedigree links). Likewise, Ba 13-13 is a genetic derivative of #169 [39]. Other relationships appear to be influenced more by geographic origin. The two *A. sterilis *genotypes diverged; however, the accession from Morocco clustered with the two Moroccan accessions of *A. magna*. Genotypes representing a unique geographic origin, such as cultivars Asencao and Kangaroo, tended to branch independently.

**Table 4:** Additional *Avena *genotypes used for SNP diversity analysis

Taxon	Identifier	Country of origin	Ploidy	Genomes
*A. strigosa*	PI 573585	Spain	Diploid	AsAs
*A. strigosa*	PI 291990	Israel	Diploid	AsAs
*A. eriantha*	Cc 7278/19	Morocco	Diploid	CpCp
*A. magna^a^*	#169 (M26)	Morocco	Tetraploid	CCDD
*A. magna*	Ba 13-13^b^	Morocco/Israel	Tetraploid	CCDD
*A. insularis*	BYU 210	Italy	Tetraploid	AACC
*A. sterilis*	PI 657407	Morocco	Hexaploid	AACCDD
*A. sterilis*	PI 411817	Iran	Hexaploid	AACCDD

^a ^Syn. *A. maroccana*

^b ^Domesticated *A. magna *[Ref. [39]]

### SNP sequence validation

**Figure 4:**
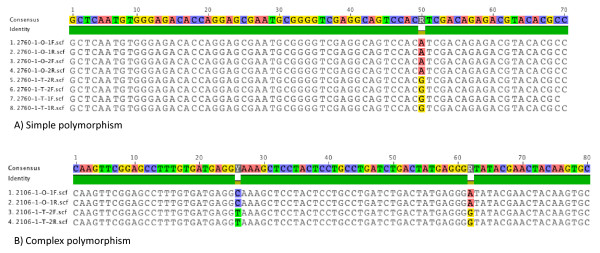
**Sequence validation**. Sequencing of real-time PCR amplicons using oat EST-SNP primers revealed both simple (A) and complex (B) polymorphism between Ogle1040 (O) and TAM O-301(T). Individual reads are identified by primer name (c2760_1 and c3768_1) and genotype (O and T). Sequences were aligned using Geneious v4.8.3.

Of the twelve primers with sequenced amplicons, seven yielded additional variations beyond what was expected from the *in silico *data: more than one base was polymorphic in one of the two cultivars (Figure 4). Since the additional variations were rare and not consistent across all reads, problems might have been due to sequencing or PCR aberrations rather than actual sequence polymorphism. An alternate explanation is that the 454 sequencing technology, which was used to discover new oat ESTs, has been associated with homopolymer errors. Our filtering methods, which excluded most in-del polymorphisms, may have excluded these instances when in fact they were real variants.

## Discussion

**Figure 5:**
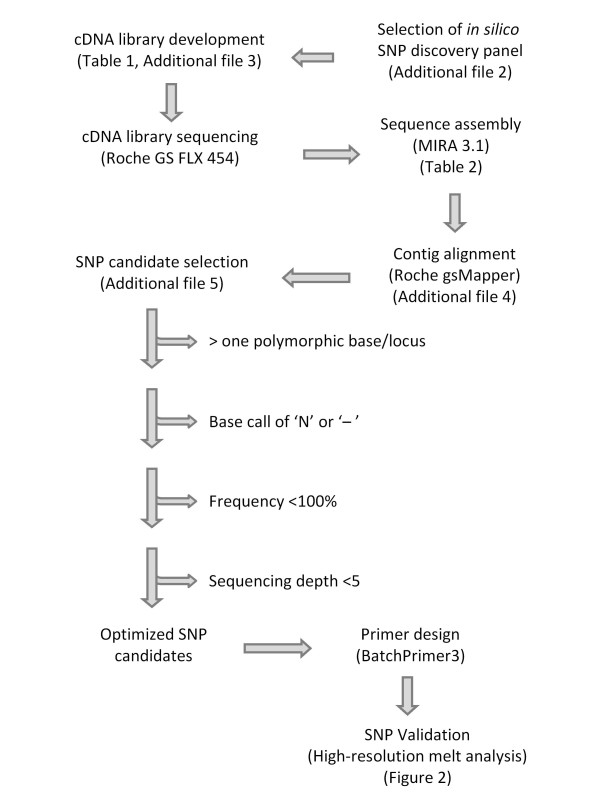
**SNP discovery pipeline**. Schematic representing a sequence for SNP discovery in a complex genome. *In silico *removal of ambiguous polymorphism was used to optimize candidate SNP sequences prior to primer design and validation.

High-throughput SNP discovery, a touchstone of modern genomics, has traditionally relied on substantial sequence data and until recently has been considered impractical for uncharacterized species, especially those with large or complex genomes. Complexities in the oat genome include paralogous and orthologous gene duplication, chromosomal rearrangements, and polyploidy. To address these difficulties, we developed a strategy integrating current-generation Roche 454 cDNA sequencing and contig assembly with novel bioinformatics, allele-calling techniques, and HRM validation (Figure 5).

Yields of RNA varied by tissue type and genotype, with variation introduced by tissue morphology and partitioning of shoot/root tissue mass in developing seedlings. Thus, mature embryo-derived RNA is represented at a three-fold level compared to root-derived RNA, with intermediate levels obtained from shoots and pistillate structures. Although tissue representation in these samples is not identical, the RNA ratios could allow backward extrapolation for research applications where tissue origin is a factor.

Variation in total RNA quantities did not appear to affect sequencing yields. TAM had the least amount of RNA, but the largest number of sequencing reads and greatest total contig length. However, TAM was the first library to be sequenced, and was used to calibrate optimal read numbers for the remaining genotype libraries. The increased read depth and coverage of TAM qualified this library as a reference genotype for sequence alignment and SNP discovery.

The use of non-normalized cDNA libraries resulted in substantial cost savings, allowing resources to be directed toward greater read depth and largely compensating for the tendency toward abundant transcripts. Additionally, the use of non-normalized libraries retained data that would be required for transcript quantification. This information, coupled with knowledge of RNA ratios derived from each tissue type, could be useful in gene expression studies, making the sequence information from these libraries valuable for a wide array of downstream research.

Contigs assembled from TAM reads were used as the reference assembly. Since TAM was one of the mapping parents used for validation, we were able to design SNP assays effectively with a high probability of success. Without using a reference assembly, many more candidates could have been incorporated erroneously into the initial design, including those categorized as ambiguously polymorphic. Furthermore, the use of a composite reference assembly could introduce additional ambiguities, since homologous loci from different cultivars would occasionally assemble into separate contigs. Alternatively, the other cultivars could be used incrementally as reference assemblies to interrogate additional loci that are absent in the first reference assembly, but this would require subsequent removal of duplicate loci. Differentiation between paralogous sequences is mainly accounted for by the parameters of the assembly, which were apparently relaxed enough to exclude non-allelic sequences from individual contigs. Screening low- and high-stringency assemblies for single-copy genes can help gauge the ability to successfully assemble known gene sets.

Proportions of SNP candidates removed during *in silico *SNP selection provided insights into specific phases of the SNP selection pipeline. Within the RA, 5% of contigs were removed due to indel polymorphism, suggesting a low rate of misassembly. Sequence quality was of greater significance: 25% of contigs were removed from the RA on account of base call ambiguity. When comparing alternate reads to the RA, 14% of contigs were removed due to indels; however, since indels were not relevant to this study, conservation within or between genotypes was not compared and thus the proportion based on misassembly is unknown. Insufficient read depth eliminated 13% of contigs, indicating adequate depth of coverage in 87% of reads and highlighting the suitability of this technology for characterization of large and complex genomes. The greatest attrition, accounting for 55% of all *in silico *SNPs, was caused by lack of SNP conservation between sequence reads of a single genotype. These ambiguities were collectively due to misassembly, which is likely to be minor based on the rate within the RA, and to sequence redundancy caused by gene duplication and subgenome homologies.

Systematic differentiation of duplicate and homoeologous loci is beyond the scope of this paper and would require sequence-based analysis or comparison of expression differences within genes. However, the substantial number of SNPs that were eliminated due to within-genotype variation does underscore the complexity intrinsic to polyploid genomes, and the necessity to account for this complexity in SNP development protocols. Previous efforts in oat marker development have always led to large proportions of ambiguous markers and/or low success rates [29-31,35]. Thus, the necessity to be selective in this work is not a surprise, but rather, an important opportunity in quality control. The recognition of subgenome homology and duplicate loci represents a fundamental challenge to *in silico *sequence analysis, but one which could facilitate efficient development of robust markers and establishment of tools for accurate subgenome dissection in species with large and complex genomes.

The large proportion of robust assays produced in this study, coupled with the clean placements of loci on the OT map and logical cluster resolution of the diversity panel, provide compelling evidence that the pipeline presented here is a useful method for global SNP discovery. However, the SNP assay validation and direct sequencing suggested that sequence polymorphism across genotypes did not consistently reflect the exact SNP identified by *in silico *methods. All SNPS were expected to follow a pattern where Ogle, Gem and HiFi contained identical alleles that differed from TAM. In Additional File 1, it is evident that almost half of the SNPS used in diversity analysis did not follow this exact pattern. This probably results from a variety of reasons: there could be variation in the genotypes used for the assay, the HRM assay may occasionally identify a non-target polymorphism, and the original sequence data may contain errors. Errors may also have been introduced during sequencing of the PCR amplicons, or through the preparatory process of cloning and purification [40].

Application of high-resolution melt analysis facilitated confirmation of informative polymorphism (Figure 2). Amplicons not corresponding to expected genotypes were easily discriminated based on the visual interface, a feature that provided ancillary information such as amplification of more than one fragment per genotype, rate and intensity of amplification, and presence of insertions and deletions within the amplicon. In the present study, this information was used to study sequence conservation across *Avena *genotypes. Using HRM, we have seen conservation within expressed genic regions of cultivated oat germplasm, and occurrence of indels in related *Avena *species. These results suggest that HRM is the technology of choice to study the evolutionary ancestry of SNP loci.

Although the primer regions were frequently conserved even across 'wild' *Avena *species, considerable diversity in amplicon sizes occurred due to insertions and deletions within the amplified fragment. These additional alleles allowed dissection of the diversity panel, with cluster separation indicated primarily by genetic ancestry and genome similarities. Unlike wheat, diploid predecessors of tetraploid and hexaploid oat have not been identified, and plasticity within genomes makes it unlikely that diploid ancestors will ever be recognized unequivocally, since extant diploids have likely diverged since oat polyploidization. However, the relationships of non-*sativa *genotypes within this study provide insight on the structure of genomes present in these species. The diploid species, *A. strigosa *(A_s_A_s_) and *A. eriantha *(C_p_C_p_) diverged from both tetraploid and hexaploid genotypes. *Avena eriantha *clustered with a major clade, although on an independent branch; however, both *A. strigosa *genotypes were disjoined from the dendrogram, suggesting dissimilarity of this genome with the A genome in tetraploid and hexaploid oat species. This result corroborates previous studies, which showed more frequent chromosomal rearrangements in the A genome, and less A-genome homology between oat species of different ploidy levels [41-43]. The tetraploid *A. magna *species (genomes CCDD) were clustered independently, between two major clades, while *A. insularis *(genomes AACC) clustered within a clade comprised of hexaploid genotypes (AACCDD), suggesting greater similarity of these genomes with the corresponding genomes of cultivated oat. Although the two *A. sterilis *(AACCDD) accessions were separated, both clustered with other hexaploid genotypes, likewise indicating similarity of the genomes.

Morphological and cytogenetic studies have suggested that *A. insularis*, rather than *A. magna*, is a probable tetraploid ancestor to cultivated oat, largely due to C-genome variations [39,44,45]. Recent studies in *A. magna *have provided further evidence for a divergent C genome, using patterns of minor allele clustering to define genome origin of individual linkage groups and to establish genome relationships within *Avena *species [unpublished data, Oliver et al.]. Given the divergence of the C genome, the sequence similarity and reassortment within the A and D genomes, and the resulting tendency to assemble composite AD contigs, it seems likely that a disproportionate number of AD SNP loci were removed due to misassembly, filtering bias, or redundant loci. Thus, the C genome could be overrepresented in these SNP markers. In genetic map construction, this bias would result in seven more densely-populated linkage groups, an outcome which is suggested, but not confirmed, with our limited data (Table 3). Future work with large-scale marker development and mapping, and physical anchoring with monosomic oat hybrids, should help to resolve this question.

Further validation of this marker development approach was provided by the linkage map positions of markers polymorphic in the OT population. Considering the small sample size, SNP markers showed remarkable coverage, mapping across 17 of the 23 existing linkage groups. Additionally, the proportion of SNP makers mapping to the framework provides internal evidence for mapping accuracy of the markers.

Several groups of SNP markers were derived from a single contig (Table 3). Of these, two pairs mapped to the same locus, likely representing haplotypes which differ at more than one SNP. Three pairs mapped to different loci on the same linkage group. Although separate map positions were not predicted for these markers, independent SNP loci with a shared contig origin could represent sequence duplication within the chromosome, a prevalent occurrence within the oat genome [46]. More predictable were groups of SNPs representing the same contig but mapping to different linkage groups. This may result from interlocus variation that was confounded with allelic variation in the *in silico *discovery process, and it underscores the need to validate all *in silico*-derived SNPs through mapping. Although this adds complexity to the process, the characterization of related loci can be useful in the discovery of alternate disease-resistance alleles or epistatically-interacting genes, and may help to define homoeologous relationships between oat chromosomes. As an example, previous work has suggested association of multiallelic SSR map positions and interacting disease resistance loci as a method for identifying potential homoeologous chromosomes [35]. In that work, SSR alleles derived from the same primer or sequence were mapped to different linkage groups. Of particular interest were two pairs of linkage groups which appeared to represent homoeologous chromosomes: OT_32_33 and OT_27, and OT_6 and OT_11, which contain alternate alleles of known resistance genes. Similar linkage group associations were identified using groups of SNP markers derived from the same contig. For example, SNP loci from RA c11164 mapped to LG_6 and LG_11, linking closely to the respective alternate QTL and homoeologous thaumatin-like protein (TLP) loci [35,47]. A less direct connection was observed using a combination of RA c2391 and RA c12516: loci from RA c2391 mapped to OT_32_33 and OT_34, and loci from RA c12516 mapped to OT_27 and OT_34. Taken together, these shared contig sequences could suggest homoeologous chromosome representations from the three oat genomes. Other data points potentially obscure the data, such as RA c2391, which mapped to OT_30 as well as OT_32_33 and OT_34. Nonetheless, the putative correlations are compelling and warrant future research to confirm these identities and establish other possible homoeologous relationships.

## Conclusions

The SNP discovery and validation pipeline presented in this study has been shown to be an effective method for identification of SNP markers in oat, a species with a complex and poorly-characterized genome. These markers had a high assay validation rate and proven utility in a variety of applications. In this study, we provide evidence that interrogation of SNPs from the same contig might allow delineation of homoeologous chromosomal relationships between genomes. Additionally, interrogation of SNP loci with HRM revealed a potential application for studying the evolutionary ancestry of the loci. Overall, this work provides the first set of oat-based SNP markers, and a pipeline for large scale development of a much-needed genomic resource. Impacts of this work will be seen in areas of QTL and association mapping, and studies of genome structure and evolution, leading to the accelerated improvement of oat through marker-assisted breeding.

## Methods

### Plant materials

Four oat cultivars were selected for cDNA library construction and sequencing (Additional file 2, rows 1-4). Contig alignments from these cultivars were used to identify candidate SNPs. Validation of SNP assays was performed using Ogle and TAM parental lines and the 136 F_6 _derived recombinant inbred lines of the OT mapping population [38]. Analysis of SNP diversity was evaluated using a panel consisting of the four genotypes used in marker development and 22 additional lines selected to represent genetic diversity in North American oat breeding germplasm (Additional file 2, rows 5-28). Selection of this material was based primarily on principal component analysis of DArT marker polymorphism [36]. Also included in the diversity panel were eight genotypes representing five different *Avena *species (Table 4). These materials were selected to represent three ploidy levels and various genome combinations, to facilitate a broader study of allele composition within *Avena*.

### RNA and DNA isolation

Total RNA for development of transcriptome libraries was isolated from four tissue types: mature embryos, pistillate structures or immature embryos, etiolated shoots, and roots (Additional file 3). Tissues were harvested from seed tracing back to a single genetic stock for each genotype. Seed for each genotype was surface-sterilized with 5% sodium hypochlorite (NaClO) and plated on moist filter paper under sterile conditions. Mature embryos were excised approximately 24-30 hours after plating, while shoots and roots were harvested after approximately five days. Plates were kept in the dark until tissue was collected. Pistillate structures were collected approximately at anthesis from surface-sterilized panicles produced in a growth chamber. Growth chamber conditions consisted of 21d at 7.2°C with a 9-h photoperiod, followed by a 30°C/21°C daily phase with a 15-h photoperiod. Harvested tissues were flash-frozen in liquid N_2 _and stored at -80°C, and RNA was extracted using an UltraClean plant RNA isolation kit (Mo Bio Laboratories, Cat. No. 13300-50) according to the manufacturer's guidelines. RNA concentration from each tissue type was determined using a NanoDrop^® ^ND-1000 spectrophotometer and pooled for each genotype, as listed in Table 1. RNA pools were frozen (-80°C) and shipped to Beckman Coulter Genomics (Beverley, MA) for cDNA library development and sequencing.

DNA for SNP validation and linkage analysis was extracted using a cetyl trimethyl ammonium bromide (CTAB) protocol. In brief, seedling leaf tissue was frozen at -80°C, ground in liquid N_2_, and incubated with 1 ml extraction buffer (0.35 M sorbitol, 0.3 M TrisHCl pH 8.0, 5 mM EDTA pH 8.0, 2 M NaCl, 2% CTAB, 5% (w/v) *N*-Lauroylsarcosine, 2% (w/v) Polyvinylpyrrolidone (PVP40, K29-32), and 0.5% (w/v) sodium metabisulfite) at 65°for one h. DNA was extracted with 24:1 chloroform:isoamyl alcohol, precipitated with isoproanol, washed with 70% EtOH, and resuspended in 10 mM Tris buffer.

### cDNA library construction

Total RNA for each genotype was isolated from pooled RNA pellets by sample lysis using TRIzol reagent and dissolved in nuclease-free water at an approximate concentration of ≥1 μg/μl. Poly(A)+RNA from total RNAs was isolated by two rounds of oligo(dT) selection with oligo(dT)-coated magnetic particles (Seradyn, Inc.). From the poly(A)+RNA, cDNA libraries were constructed using an oligo dT primer-adapter containing a *Not*I site and Moloney Murine Leukemia Virus Reverse Transcriptase (M-MLV RT) to prime and synthesize first strand cDNA. After second strand synthesis, double-stranded (ds) cDNA was size fractionated (>1.2 kb) and cloned directionally into the *Not*I and *Eco*RV sites of the pExpress 1 vector. Primary clones were produced for each genotype from one bulk ligation (300 ng pExpress 1 vector, *Not*I-*Eco*RV cut, and 120 ng of *Not*I-digested cDNA per 120 μl ligation) followed by electroporation into T1 phage resistant *E. coli*.

### Roche 454-Ready cDNA and sequencing

The four synthesized library DNAs were digested with *Not*I, and *in vitro *RNA transcripts were produced using the SP6 RNA polymerase promoter. First strand cDNA was made from these transcripts using a modified primer adapter that reduces the size of the poly(A) sequence to about 20 As. After synthesis of the second strand, ds cDNA was blunt ended and size fractionated. This ds cDNA was resuspended in TE, pH 8.0, to between 110-125 ng/μl. Published Roche 454 GS FLX-Titanium protocols were followed for sequencing. In brief, 3-5 μg DNA was nebulized to a mean size range of 40-800 bp, followed by size selection of fragments >300 bp by column exclusion and Ampure™ (Agencourt Bioscience) isolation. Correct size selection was confirmed on an Agilent DNA 1000 LabChip. Adapters were ligated onto the fragments and fragments with correct adapters were selected using library capture beads. Single stranded fragments were isolated with 0.125 N NaOH, neutralized with acetic acid, and purified. Single stranded libraries were validated qualitatively by the Agilent RNA Pico 6000 LabChip and quantitatively by the Invitrogen Ribogreen assay. Standard library dilutions were made according to the published protocol.

Each library was amplified onto DNA capture beads by emulsion PCR (emPCR). DNA capture beads were collected by washes with isopropanol, Roche 454 emPCR collection reagents, and filtered syringes. Sequencing primer was annealed by thermocycling and collected beads were quantified by counting on a Beckman Multisizer. Beads for each genome were placed on the picotitre plate and sequenced on a Roche 454 GS FLX instrument. Associated image analysis and base-calling software were performed with standard protocols and default parameters. 454 reads were generated using the Roche 454 GS-FLX Sequencer.

### Sequence assembly and annotation

Sequence assembly was performed using the MIRA assembly program [[48], http://sourceforge.net/projects/mira-assembler] version 3.1 with 454 EST assembly specific parameters. The MIRA 3.1 assembly software was used in place of the Roche 454 Newbler (gsAssembler) version 2.0 based on flexibility of the parameter conditions which were part of the software package; current versions of the Roche gsAssembler now have added parameter selection and generate assemblies comparable to those of the MIRA version used. Of the four genotypes sequenced, TAM had the greatest read depth and was therefore used as a reference for base calling of candidate SNPs.

Each contig was searched against the UniProt Knowledgebase (Release 15.13, Jan 2010) using BLASTX, and matches which were≤E-10 and at least 30% similar were compared to annotated terms of the Gene Ontology (GO) Consortium [49]. Using GO/UniProt comparison tables (gene_association.goa_uniprot, Apr 2010), candidate GO assignments were predicted on the basis of contig matches to the UniProt reference sequences. Categories were assigned on the basis of biological, functional, and molecular annotations available from GO. Molecular GO terms were compared between genotypes using a four-way Venn diagram constructed using Venny [50].

### *in silico *SNP identification

The Roche gsMapper software was run for all Roche 454 sequencing runs against the reference contig assembly. Candidate SNPs were selected based mainly on header file data, which included contig name, nucleotide start and end position, reference and polymorphic base, variation frequency, and sample depth or redundancy (Additional file 4). Since the software called all sequence differences between the reference and other genotypes, removal of ambiguous polymorphism was required to optimize SNP calls. *in silico *SNPs were removed for: 1) nucleotide variations greater than one base; this excluded regions of complex polymorphism, 2) nucleotide variations designating an "N" or "-"; this excluded non-uniform polymorphism and insertion/deletion, 3) nucleotide variations with a frequency less than 100% within a genotype; this avoided non-uniform polymorphism, 4) nucleotide variations with a sequencing depth less than five; this assured accuracy (Additional file 5).

### SNP assay design, validation, and diversity analysis

Contigs containing *in silico *SNPs were upload into BatchPrimer v1.0 http://avena.pw.usda.gov/demos/batchprimer3 following the NCBI dbSNP FASTA format with SNP alleles masked using the IUB/IUPAC nucleic acid code [37]. Primers were picked using the "Single base extension (SBE) primers and SNP (allele) flanking primers" type. Default settings were used to select both the SNP-specific and flanking primers with the only exception being an optimal product size of 90 bp +/- 30 bp. Likewise, default settings were used for penalty weights. Assays for which both flanking primers and forward and reverse orientation SNP primers could be designed were considered successful and validated (Additional file 6).

Assay validation was performed using high-resolution melt (HRM) analysis of real-time PCR products. The reaction comprised 1× SsoFast EvaGreen Supermix (BioRad, #172-5201) with 55 ng genomic DNA and 0.5 μM forward and reverse primers in a 12.5 μl reaction volume. Thermocycling was performed in 96-well PCR microplates on a BioRad C1000 thermal cycler with a CFX96 optics module, using the following reaction conditions: initial denaturation at 98°C for 2 min; 46 cycles of 98°C for 2 s and 55°C for 5 s, with a fluorescent reading taken at the end of each cycle; and a melt curve analysis, with a melt gradient from 65°C to 95°C, increasing in 0.2°C increments every 10 s, with a fluorescent reading taken at the end of each increment. The HRM analyses were carried out using BioRad Precision Melt Analysis Software Version 1.0.534.0511. Genotypes were assigned by examining the difference and melt curves, based on relative fluorescence units (RFU) as a function of melting temperature. Map positions of markers polymorphic in the OT population were assigned by appending new loci to the existing OT linkage map [35] using Map Manager QTX [51].

High-resolution melt analysis was used to evaluate 33 SNP markers across the diversity panel. For each marker, genotypes not corresponding to the bi-allelic model were noted and scored as an insertion (larger difference RFU), deletion (smaller difference RFU), or heterozygous (intermediate between standard genotypes), according to the difference plot.

To study genetic structure and relationships within the diversity panel, genetic similarities were estimated for the oat lines using Dice's index [52] with NTSYSpc ver. 2.20q [53]. Prior to analysis, the allele of each SNP locus and/or presence or absence of an indel was coded as one or zero to produce a binary data matrix. From the similarity matrix, a dendrogram was constructed using the unweighted pair group method with averages.

### SNP sequence validation

To validate SNP sequence, DNA of Ogle and TAM was PCR amplified, using the same reaction and thermocycling conditions as for SNP validation. PCR products were separated on 1% agarose, and DNA bands were cut from the gel and dissolved in elution buffer (10 mM magnesium acetate tetrahydrate, 0.5 M ammonium acetate, 1 mM EDTA, and 0.1% (w/v) SDS). Gel fragments were incubated overnight at 37°C, and DNA was purified from the solution using 24:1 chloroform:isoamyl alcohol, washed twice in 70% EtOH, and resuspended in H_2_O. Resuspended products were used as a template to reamplify the fragment, and products were checked on an agarose gel.

Gel-purified DNA was transformed into the pGEM^®^-T Easy Vector System using JM109 competent cells, following the manufacturer's protocol (Promega, Madison, WI, USA). DNA was extracted from the plasmid using the GenElute Plasmid Miniprep kit (Sigma, St. Louis, MO, USA) and 300-400 ng plasmid DNA was amplified using Big Dye cycle sequencing and M13 forward (5' GTA AAA CGA CGG CCA GT 3') and reverse (5' CAG GAA ACA GCT ATG AC 3') primers. The sequencing reaction profile included 25 cycles of 96°C for 10 s followed by 50°C for 6 s, and 60°C for 4 min. Amplified PCR product was purified with Sephadex G-50 (GE Healthcare) and sequenced with an ABI3730xl DNA Analyzer (Applied Biosystems, Foster City, California). Sequenced vectors were screened using NCBI VecScreen and the region of interest was confirmed by BLASTing each sequence against the NR database in NCBI. Sequences which were conserved with mRNA, cDNA clones, and hypothetical proteins of grass families were taken as the insert of interest and included in this study. Sequences were aligned using Geneious v4.8.3 [54].

## Authors' contributions

EWJ and REO led the design and coordination of this study, prepared RNA and DNA samples, implemented HRM, mapped loci, analyzed overall data, and drafted the initial manuscript. GRL played a major role in planning this study, led the bioinformatics pipeline development and conducted all related analyses, drafted some sections of the manuscript, and prepared some tables and figures. MJR supervised cDNA library construction, Roche 454 sequencing, and contig assembly. NAT assisted in research design and overall data analysis, and made substantial contributions to the bioinformatics pipeline and drafting of the manuscript. EWJ, REO, JDL, GBG, SC, JMA, ENJ, and PJM assisted GRL and NAT in development of the bioinformatics pipeline. NHWM assisted in SNP validation and diversity analyses. JMB analyzed diversity data and constructed the dendrogram. DA carried out sequence validation at Brigham Young University and ENJ and PJM assisted in analysis and interpretation of results. Most of the above, and all additional authors, contributed to the inception, design, and interpretation of this work. All authors reviewed and approved this manuscript.

## Supplementary Material

Additional file 1 **Diversity panel allele designations**. Allele calls were based on high-resolution melting analysis of PCR amplicons generated with 36 EST-SNP markers in a panel of 34 diverse oat genotypes. Supplementary alleles were designated as insertion (In), deletion (Del), or null (no amplification). When SNP sequences were not available, alternate alleles were designated X and Y. Click here for file

Additional file 2 **Origin and pedigree links of oat genotypes used in SNP development, validation, and diversity analysis**. The first four genotypes were used in cDNA library construction and sequencing and SNP marker discovery. All genotypes in this table, together with the additional genotypes listed in Table 4, were used for SNP genotyping and diversity analysis. Click here for file

Additional file 3 **Tissue types**. RNA was extracted from etiolated shoots (A) and roots (B), pistillate structures (C), and mature embryos (D) from four different oat varieties. Tissues were grown at standardized conditions, and RNA for each tissue type was extracted at the same stage of development. Click here for file

Additional file 4 **Header report files generated by Roche gsMapper**. Powerpoint file displaying header report files generated by Roche gsMapper. Click here for file

Additional file 5 **Command line processing of header files and sequences for SNP candidate design**. Powerpoint file displaying command line processing of header files and sequences for SNP candidate design. Click here for file

Additional file 6 **Primer and allele sequences of SNP markers mapped in the Ogle1040/TAM O-301 RIL population**. Word DOC file displaying primer and allele sequences of SNP markers mapped in the Ogle1040/TAM O-301 RIL population. Click here for file
