# Supplementary material for: Model SNP development for complex genomes based on hexaploid oat using high-throughput 454 sequencing technology
Source: BMC Genomics. 2011 Jan 27;12:77. doi: 10.1186/1471-2164-12-77 (PMC3041746; doi:10.1186/1471-2164-12-77)
Supplement: Additional file 1 — Diversity panel allele designations. Allele calls were based on high-resolution melting analysis of PCR amplicons generated with 36 EST-SNP markers in a panel of 34 diverse oat genotypes. Supplementary alleles were designated as insertion (In), deletion (Del), or null (no amplification). When SNP sequences were not available, alternate alleles were designated X and Y. [file 1471-2164-12-77-S1.DOC]

**Additional file 6: Diversity panel allele designations.** Allele calls were based on high-resolution melting analysis of PCR amplicons generated with 36 EST-SNP markers in a panel of 34 diverse oat genotypes. Supplementary alleles were designated as insertion (In), deletion (Del), or null (no amplification). When SNP sequences were not available, alternate alleles were designated X and Y.

|  | **SNP marker** | | | | | | | | | | | | | |
| --- | --- | --- | --- | --- | --- | --- | --- | --- | --- | --- | --- | --- | --- | --- |
| **Genotype** | **c51_2** | **c75_1** | **c96_1** | **c104_1.82** | **c104_1.86** | **c236_2** | **c236_3** | **c250_1** | **c557_1** | **c841_2** | **c841_3** | **c876_1** | **c1361_1** | **c2043_1** |
| Noble-2 | C | T | C | Y | Y | C | C | A | Y | T | T | A | C | C |
| MN 84 | C | A | C | Y | X | G | C | G | Y | G | T | A | A | G |
| TAM 405 | T | A | T | X | Y | C | C | G | Y | T | G | In | A | C |
| Otana | C | T | T | Y | Y | G | T | A | Y | G | T | T | A | C |
| Sun II | C | T | T | Y | X | G | T | G | X | T | G | T | A | C |
| Hurdal | In | A | T | Y | X | G | T | G | X | G | T | T | A | G |
| AC Morgan | C | A | T | Y | Y | G | T | G | X | T | G | T | A | G |
| Goslin | C | A | T | Y | Y | C | C | A | Y | T | T | A | C | C |
| Asencao | C | T | C | Y | X | C | C | A | Y | T | T | A | C | G |
| Ajay | C | T | C | X | X | C | T | A | Y | T | T | A | A | C |
| Ogle | C | T | C | Null | Y | C | C | A | Y | T | T | A | C | C |
| AC Rigadon | C | T | C | X | X | C | T | A | Y | T | T | A | C | C |
| Gem | C | T | C | Null | Y | C | C | A | Y | T | T | A | C | C |
| AC Marie | C | T | T | X | Y | C | T | A | Y | G | Het | T | A | G |
| CDC Dancer | T | T | T | Y | X | C | T | A | Y | T | Het | T | C | G |
| Assiniboia | C | T | T | Y | X | C | T | G | X | T | G | A | A | G |
| Buckskin | T | A | T | Y | X | C | C | A | X | G | Het | T | A | G |
| HiFi | C | T | C | Y | Y | C | C | A | X | G | T | A | C | G |
| Tam O-301 | T | A | T | Y | X | C | T | G | X | G | G | T | - | G |
| Coker 227 | C | A | C | Y | Y | G | C | G | X | T | G | A | A | C |
| Kanota | T | A | C | Y | Y | G | C | G | Y | T | T | A | - | G |
| Kangaroo | Del | T | T | X | X | C | C | A | Y | In | Het | A | C | G |
| CI 4706-2 | Del | T | C | Null | Het | C | C | A | Y | Del | T | A | C | C |
| Tardis | C | A | - | Null | Y | G | T | G | X | T | G | A | - | G |
| Buffalo | C | T | C | X | Y | C | T | A | Y | G | T | A | C | G |
| Maverick | C | T | T | X | Y | C | C | A | Y | G | T | In | A | C |
| Ba 13-13 | In | Del | T | Null | Y | In | C | A | Y | In | T | A | C | Het |
| #169 | Del | Del | C | Null | Y | In | C | A | Y | In | Het | A | C | Het |
| BYU 210 | In | T | C | Y | Het | C | C | A | Y | T | G | In | C | Het |
| Cc 7278 | C | A | T | Null | X | In | In | In | X | In | Het | A | A | In 1 |
| PI 291990 | Del | - | In | Het | X | In | In | In | Null | Del | Del | A | C | C |
| PI 411817 | Del | T | In | Y | X | C | C | A | X | G | T | A | C | G |
| PI 573585 | Del | - | In | Het | X | In | In | In | Null | Del | Del | Del | Del | In 2 |
| PI 657407 | T | A | T | Null | Y | G | C | A | X | Het | A | C | C | C |

|  | **SNP marker** | | | | | | | | | | | | | | | | | | | | | |  |
| --- | --- | --- | --- | --- | --- | --- | --- | --- | --- | --- | --- | --- | --- | --- | --- | --- | --- | --- | --- | --- | --- | --- | --- |
| **Genotype** | **c2391_4** | **c2391_5** | | **c2539_1** | | **c2680_1** | | **c2760_1** | | **c4096_1** | | **c5153_1** | | **c5252_1** | **c11164_2** | | **c12344_1** | | **c12516_2** | | **c14374_2** | |  |
| Noble-2 | G | T | | Y | | C | | Het | | T | | G | | G | Y | | G | | A | | C | |  |
| MN 84 | G | T | | X | | T | | Het | | T | | A | | G | Y | | In | | In | | T | |  |
| TAM 405 | C | G | | X | | C | | Het | | T | | G | | G | Y | | G | | A | | C | |  |
| Otana | G | T | | X | | C | | T | | C | | A | | G | Y | | C | | A | | C | |  |
| Sun II | G | T | | Y | | Het | | T | | C | | A | | A | X | | C | | A | | T | |  |
| Hurdal | G | T | | Y | | T | | In | | T | | A | | G | X | | G | | Del | | In | |  |
| AC Morgan | G | Del | | Y | | C | | Het | | T | | G | | A | X | | G | | A | | C | |  |
| Goslin | C | G | | Y | | T | | T | | T | | G | | G | Y | | C | | A | | T | |  |
| Asencao | G | T | | X | | C | | T | | C | | A | | G | Y | | C | | G | | T | |  |
| Ajay | G | T | | Y | | C | | G | | C | | G | | A | Y | | C | | G | | C | |  |
| Ogle | C | G | | X | | T | | T | | C | | A | | G | Y | | C | | G | | C | |  |
| AC Rigadon | G | T | | X | | Het | | In | | T | | G | | A | Y | | C | | G | | C | |  |
| Gem | C | G | | X | | C | | T | | C | | G | | G | Y | | G | | G | | C | |  |
| AC Marie | Del | T | | Y | | C | | G | | T | | G | | A | Del | | G | | Del | | T | |  |
| CDC Dancer | G | Del | | Y | | T | | G | | T | | G | | A | X | | C | | Del | | C | |  |
| Assiniboia | G | T | | Y | | C | | T | | C | | G | | G | X | | G | | A | | T | |  |
| Buckskin | G | T | | Y | | C | | T | | C | | G | | A | X | | G | | G | | C | |  |
| HiFi | C | G | | Y | | C | | Het | | T | | G | | G | X | | G | | Del | | C | |  |
| Tam O-301 | G | T | | X | | T | | G | | T | | G | | A | X | | G | | A | | T | |  |
| Coker 227 | C | G | | Y | | T | | T | | T | | G | | A | X | | C | | In | | C | |  |
| Kanota | C | G | | X | | C | | T | | C | | A | | A | Y | | C | | In | | C | |  |
| Kangaroo | C | G | | In | | C | | T | | C | | G | | A | X | | In | | In | | C | |  |
| CI 4706-2 | C | G | | In | | C | | Null | | C | | G | | G | Del | | Del | | G | | T | |  |
| Tardis | G | T | | X | | C | | G | | T | | A | | A | Y | | G | | G | | T | |  |
| Buffalo | G | Del | | X | | C | | G | | T | | Del | | A | Del | | G | | A | | C | |  |
| Maverick | G | T | | X | | C | | G | | C | | G | | A | Y | | In | | G | | C | |  |
| Ba 13-13 | C | G | | In | | C | | In | | Del 1 | | A | | In | In | | Del | | G | | T | |  |
| #169 | C | T | | In | | C | | In | | C | | A | | In | In | | Del | | G | | T | |  |
| BYU 210 | C | G | | X | | C | | T | | T | | G | | In | Y | | Del | | G | | C | |  |
| Cc 7278 | In | G | | Y | | C | | In | | Del 1 | | A | | G | Null | | In | | Null | | Del | |  |
| PI 291990 | In | In | | Del | | In | | Null | | Del 1 | | Null | | G | Y | | Del | | G | | In | |  |
| PI 411817 | C | T | | Y | | C | | Het | | T | | G | | Del | X | | Del | | G | | T | |  |
| PI 573585 | In | In | | Del | | In | | Null | | Del 1 | | Null | | Y | Del | | Del | | A | | In | |  |
| PI 657407 | G | Del | | Y | | Het | | Null | | Del 2 | | A | | X | Del | | Del | | T | | X | |  |
|  |  |  | |  | |  | |  | |  | |  | |  |  | |  | |  | |  | |  |
|  | **SNP marker** | | | | | | | | | | | | | | | | | | | | | | |
| **Genotype** | **c14852_1** | | **c14852_2** | | **c15098_1** | | **c16908_1** | | **c22314_1** | | **lrc16053_1** | | **lrc16053_2** | | | **lrc16053_3** | | **lrc21826_1** | | **lrc34490_1** | | **lrc38531_2** | |
| Noble-2 | G | | A | | 1 | | G | | G | | A | | X | | | T | | G | | C | | X | |
| MN 84 | G | | A | | 1 | | Del | | Del | | A | | Y | | | C | | T | | C | | Y | |
| TAM 405 | T | | C | | 2 | | G | | G | | G | | X | | | C | | G | | A | | Y | |
| Otana | G | | A | | 1 | | G | | T | | G | | In | | | C | | T | | C | | X | |
| Sun II | G | | A | | 1 | | G | | T | | G | | X | | | C | | G | | A | | Y | |
| Hurdal | G | | A | | 1 | | G | | T | | G | | X | | | C | | T | | A | | X | |
| AC Morgan | G | | A | | 2 | | G | | T | | G | | X | | | C | | G | | A | | X | |
| Goslin | T | | C | | 2 | | G | | G | | G | | In | | | T | | G | | A | | Y | |
| Asencao | G | | A | | 1 | | G | | G | | A | | In | | | T | | G | | C | | Y | |
| Ajay | T | | C | | 1 | | G | | T | | A | | Y | | | T | | G | | C | | Y | |
| Ogle | T | | C | | In | | G | | G | | A | | X | | | T | | T | | A | | Y | |
| AC Rigadon | G | | A | | 2 | | G | | T | | G | | X | | | C | | G | | A | | X | |
| Gem | T | | C | | 2 | | G | | G | | A | | X | | | T | | T | | A | | Y | |
| AC Marie | G | | A | | 1 | | G | | T | | G | | X | | | C | | G | | A | | X | |
| CDC Dancer | G | | C | | 2 | | G | | T | | G | | X | | | C | | G | | A | | X | |
| Assiniboia | G | | A | | 1 | | G | | T | | G | | X | | | C | | G | | C | | Y | |
| Buckskin | T | | C | | 1 | | G | | T | | G | | X | | | C | | G | | A | | X | |
| HiFi | G | | A | | 2 | | G | | T | | A | | Y | | | T | | G | | A | | X | |
| Tam O-301 | G | | A | | 2 | | Del | | T | | G | | Y | | | C | | T | | C | | - | |
| Coker 227 | T | | C | | In | | G | | Del | | A | | Y | | | C | | T | | C | | X | |
| Kanota | G | | C | | In | | Del | | G | | A | | In | | | C | | T | | C | | Y | |
| Kangaroo | T | | C | | In | | Del | | T | | A | | Del | | | C | | T | | A | | X | |
| CI 4706-2 | T | | C | | 1 | | G | | G | | A | | Y | | | T | | G | | A | | Del | |
| Tardis | T | | C | | 1 | | G | | T | | G | | In | | | C | | T | | A | | X | |
| Buffalo | G | | A | | 2 | | G | | T | | A | | X | | | C | | G | | C | | X | |
| Maverick | T | | C | | 1 | | G | | Del | | A | | Del | | | T | | G | | C | | Y | |
| Ba 13-13 | T | | C | | 2 | | G | | T | | A | | In | | | T | | Del | | C | | Y | |
| #169 | T | | C | | 2 | | G | | In | | A | | In | | | C | | Del | | C | | Del | |
| BYU 210 | T | | C | | 2 | | G | | In | | A | | In | | | C | | T | | C | | Del | |
| Cc 7278 | T | | C | | 2 | | G | | In | | In | | Y | | | In | | Del | | Del | | In | |
| PI 291990 | Del | | C | | In | | Null | | Null | | In | | Null | | | C | | In | | C | | Null | |
| PI 411817 | T | | C | | 2 | | G | | T | | G | | In | | | T | | T | | C | | X | |
| PI 573585 | Del | | C | | In | | Null | | Null | | In | | Null | | | G | | In | | C | | Null | |
| PI 657407 | T | | C | | 2 | | G | | In | | A | | C | | | G | | C | | X | | Null | |
