# Supplementary material for: Model SNP development for complex genomes based on hexaploid oat using high-throughput 454 sequencing technology
Source: BMC Genomics. 2011 Jan 27;12:77. doi: 10.1186/1471-2164-12-77 (PMC3041746; doi:10.1186/1471-2164-12-77)
Supplement: Additional file 2 — Origin and pedigree links of oat genotypes used in SNP development, validation, and diversity analysis. The first four genotypes were used in cDNA library construction and sequencing and SNP marker discovery. All genotypes in this table, together with the additional genotypes listed in Table 4, were used for SNP genotyping and diversity analysis. [file 1471-2164-12-77-S2.DOC]

**Additional file 1: Origin and pedigree links of oat genotypes used in SNP development, validation, and diversity analysis.**  The first four genotypes were used in cDNA library construction and sequencing and SNP marker discovery. All genotypes in this table, together with the additional genotypes listed in Table 4, were used for SNP genotyping and diversity analysis.

| Genotype | Origin | Pedigree data |
| --- | --- | --- |
| Ogle | Univ. Illinois, USA | <http://avena.agr.gc.ca/OGIS/EntryShow_e.php?GID=66&NGen=3&METHOD=ancestry> |
| TAM O-301 | Texas A&M Univ., USA | <http://avena.agr.gc.ca/OGIS/EntryShow_e.php?GID=2221&NGen=3&METHOD=ancestry> |
| Gem | Wisconsin Ag. Exp. Stn., USA | <http://avena.agr.gc.ca/OGIS/EntryShow_e.php?GID=2661&NGen=3&METHOD=ancestry> |
| HiFi | North Dakota State Univ., USA | <http://avena.agr.gc.ca/OGIS/EntryShow_e.php?GID=7407&NGen=3&METHOD=ancestry> |
| Sun II-1 | Malmohus | <http://avena.agr.gc.ca/OGIS/EntryShow_e.php?GID=5238&NGen=3&METHOD=ancestry> |
| Hurdal | NULS, Norway | <http://avena.agr.gc.ca/OGIS/EntryShow_e.php?GID=18034&NGen=3&METHOD=ancestry> |
| AC Morgan | AAFC Lacombe, Canada | <http://avena.agr.gc.ca/OGIS/EntryShow_e.php?GID=3946&NGen=3&METHOD=ancestry> |
| Goslin | AAFC Ottawa, Canada | <http://avena.agr.gc.ca/OGIS/EntryShow_e.php?GID=363&NGen=3&METHOD=ancestry> |
| Asencao | Brazil | <http://avena.agr.gc.ca/OGIS/EntryShow_e.php?GID=5341&NGen=3&METHOD=ancestry> |
| Ajay | USDA ARS Aberdeen, ID, USA | <http://avena.agr.gc.ca/OGIS/EntryShow_e.php?GID=7909&NGen=3&METHOD=ancestry> |
| AC Rigodon | AAFC Sainte-Foy, Canada | <http://avena.agr.gc.ca/OGIS/EntryShow_e.php?GID=13&NGen=3&METHOD=ancestry> |
| AC Marie | AAFC Winnipeg, Canada | <http://avena.agr.gc.ca/OGIS/EntryShow_e.php?GID=3945&NGen=3&METHOD=ancestry> |
| CDC Dancer | Univ. Saskatchewan, Canada | <http://avena.agr.gc.ca/OGIS/EntryShow_e.php?GID=3956&NGen=3&METHOD=ancestry> |
| Assiniboia | AAFC Winnipeg, Canada | <http://avena.agr.gc.ca/OGIS/EntryShow_e.php?GID=2663&NGen=3&METHOD=ancestry> |
| Buckskin | Univ. Illinois, USA | <http://avena.agr.gc.ca/OGIS/EntryShow_e.php?GID=14795&NGen=3&METHOD=ancestry> |
| Coker 227 | Coker’s Pedigreed Seed, USA | <http://avena.agr.gc.ca/OGIS/EntryShow_e.php?GID=5460&NGen=3&METHOD=ancestry> |
| Kanota | Kansas State Univ., USA | <http://avena.agr.gc.ca/OGIS/EntryShow_e.php?GID=5389&NGen=3&METHOD=ancestry> |
| Kangaroo | Adelaide, South Australia | <http://avena.agr.gc.ca/OGIS/EntryShow_e.php?GID=15139&NGen=3&METHOD=ancestry> |
| CI 4706-2 | Buenos Aires, Argentina | <http://www.ars-grin.gov/cgi-bin/npgs/acc/display.pl?1029093> |
| Tardis | IGER, Aberystwyth, UK | <http://avena.agr.gc.ca/OGIS/EntryShow_e.php?GID=16721&NGen=3&METHOD=ancestry> |
| Buffalo | IGER, Aberystwyth, UK | <http://avena.agr.gc.ca/OGIS/EntryShow_e.php?GID=7481&NGen=3&METHOD=ancestry> |
| Maverick | USDA ARS Aberdeen, ID, USA | <http://jpr.scijournals.org/cgi/content/full/2/3/194> |
| MN841801 | Univ. Minnesota, USA | <http://avena.agr.gc.ca/OGIS/EntryShow_e.php?GID=8656&NGen=3&METHOD=ancestry> |
| Noble-2 | Univ. Minn. and USDA-ARS, USA | <http://avena.agr.gc.ca/OGIS/EntryShow_e.php?GID=15154&NGen=3&METHOD=ancestry> |
| TAM O-405 | Texas A&M Univ., USA | <http://avena.agr.gc.ca/OGIS/EntryShow_e.php?GID=18006&NGen=3&METHOD=ancestry> |
| Otana | USDA ARS Aberdeen, ID, USA | <http://avena.agr.gc.ca/OGIS/EntryShow_e.php?GID=3990&NGen=3&METHOD=ancestry> |
