# Supplementary figures and images for: Model SNP development for complex genomes based on hexaploid oat using high-throughput 454 sequencing technology
Source: BMC Genomics. 2011 Jan 27;12:77. doi: 10.1186/1471-2164-12-77 (PMC3041746; doi:10.1186/1471-2164-12-77)

## Slide 1
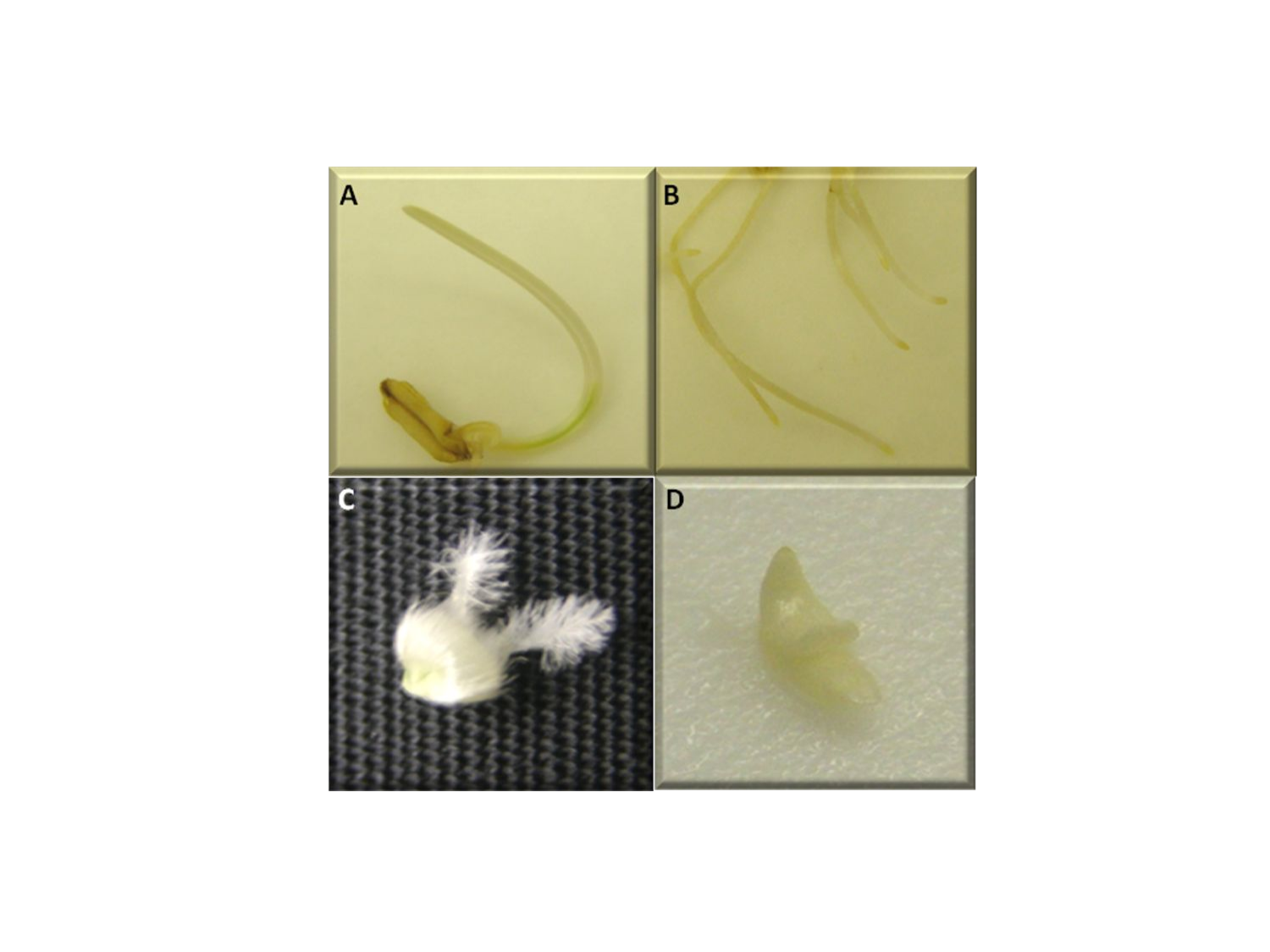

Supplement: Additional file 3 — Tissue types. RNA was extracted from etiolated shoots (A) and roots (B), pistillate structures (C), and mature embryos (D) from four different oat varieties. Tissues were grown at standardized conditions, and RNA for each tissue type was extracted at the same stage of development. [file 1471-2164-12-77-S3.PPT]

## Slide 1
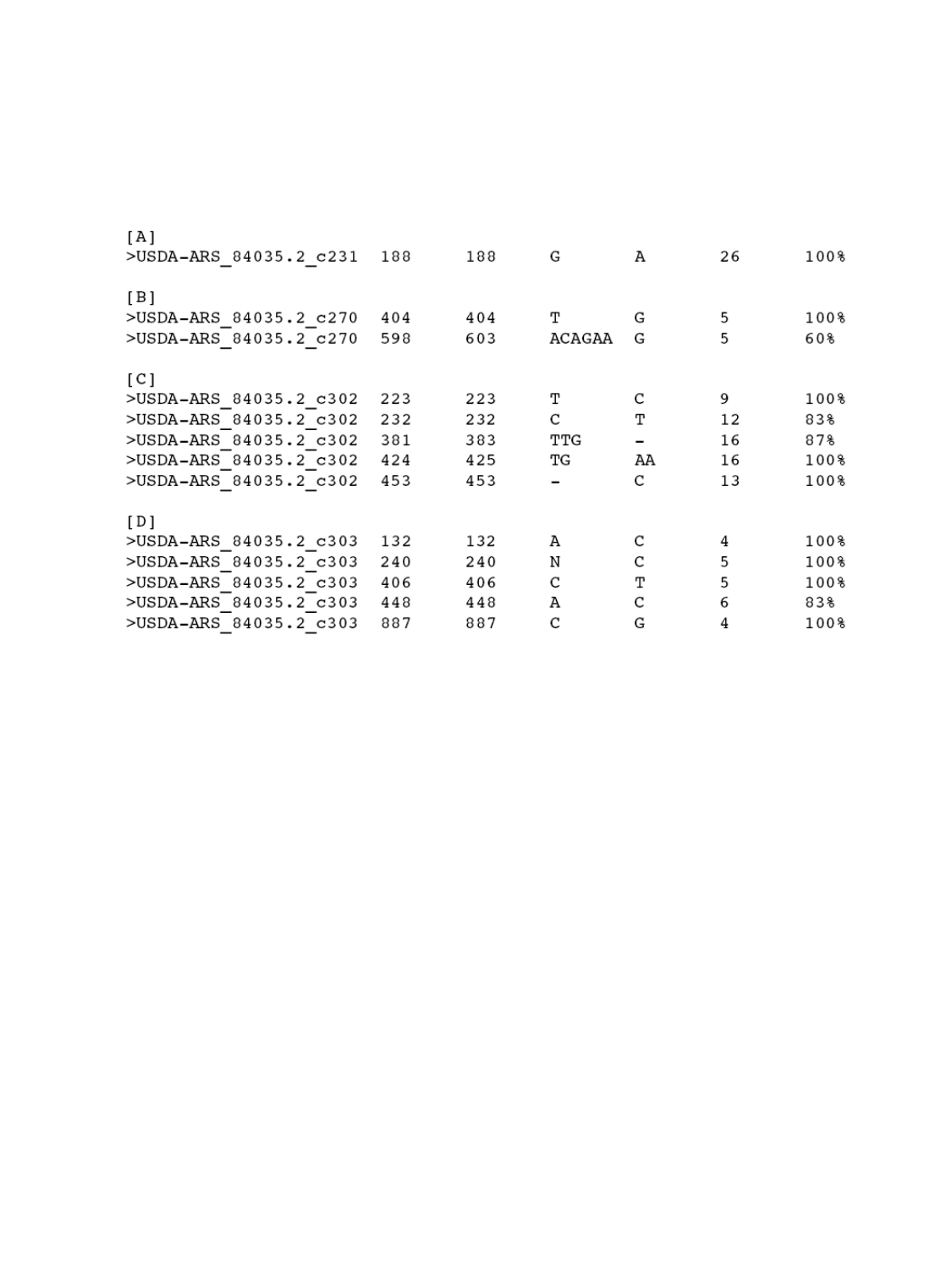

Supplement: Additional file 4 — Header report files generated by Roche gsMapper. Powerpoint file displaying header report files generated by Roche gsMapper. [file 1471-2164-12-77-S4.PPT]

## Slide 1
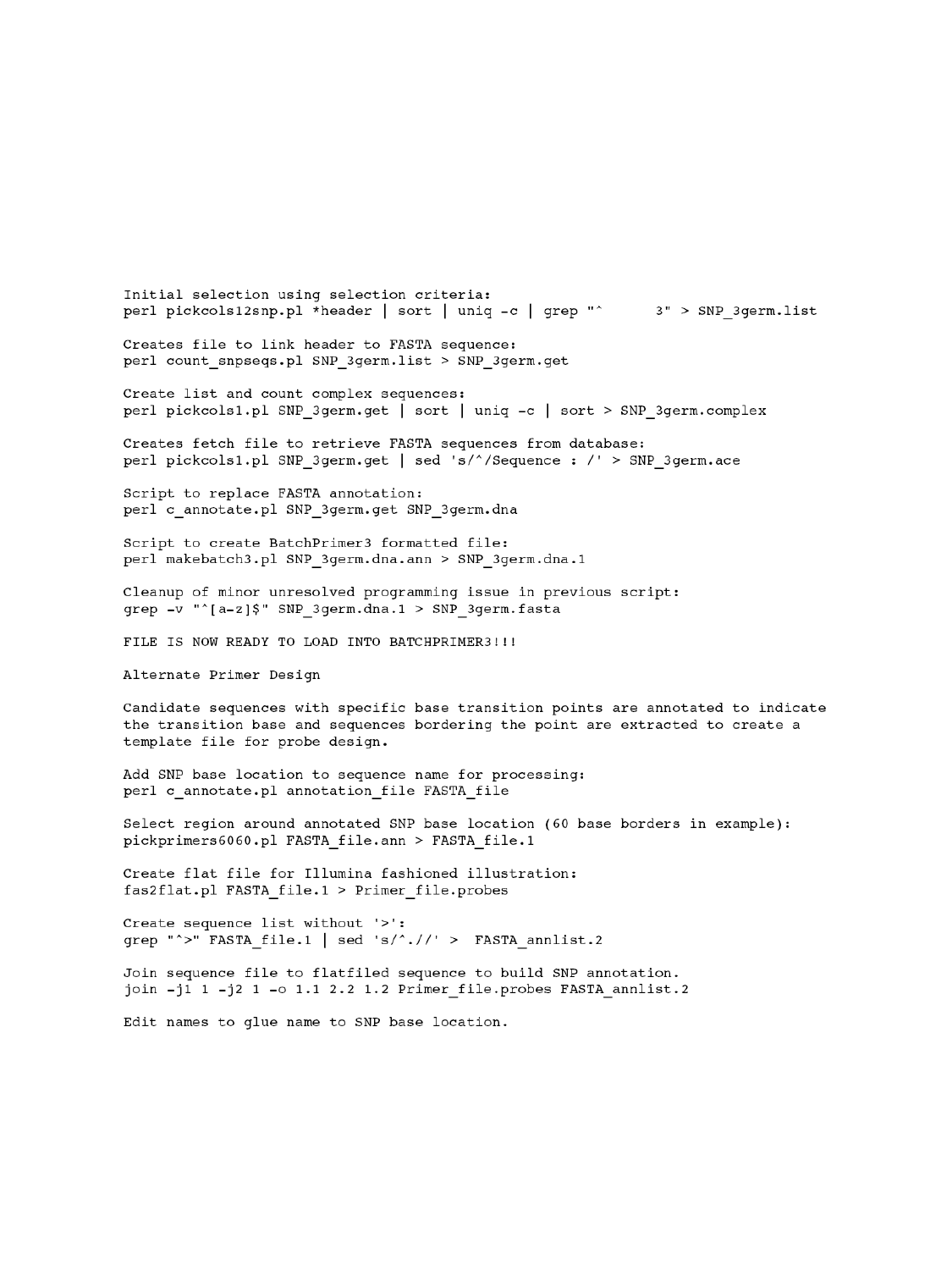

Supplement: Additional file 5 — Command line processing of header files and sequences for SNP candidate design. Powerpoint file displaying command line processing of header files and sequences for SNP candidate design. [file 1471-2164-12-77-S5.PPT]
