# Supplementary material for: Model SNP development for complex genomes based on hexaploid oat using high-throughput 454 sequencing technology
Source: BMC Genomics. 2011 Jan 27;12:77. doi: 10.1186/1471-2164-12-77 (PMC3041746; doi:10.1186/1471-2164-12-77)
Supplement: Additional file 6 — Primer and allele sequences of SNP markers mapped in the Ogle1040/TAM O-301 RIL population. Word DOC file displaying primer and allele sequences of SNP markers mapped in the Ogle1040/TAM O-301 RIL population. [file 1471-2164-12-77-S6.DOC]

Primer Name Primer Sequence SNP

oSNP_c51_1F TTCCCTGTCCAGTATGATGATG

oSNP_c51_1R TGGTACTTGCCCACACACC T/C

oSNP_c51_2F ATATGGTCGGTGGGGTGTTA

oSNP_c51_2R ACTCTCGTTGCCAAAACCAT T/C

oSNP_c104_1F TCCTCCGGTACGAGCAGAT

oSNP_c104_1R AAGGGCACTCCGAGGAAC G/A

oSNP_c250_1F CTTCTTCCTCGGAGGCTGTT

oSNP_c250_1R CCACCATGAGGGCTCGAC G/A

oSNP_c318_1F ACCAACTTCACGGTGTTCG

oSNP_c318_1R ATCTGCAGGAACGCGAAG G/C

oSNP_c540_1F GCAGCTTCCAACACCAGATT

oSNP_c540_1R AAAACCGGAGCCAACACAT G/A

oSNP_c841_2F GTGGATTGGTGCGTTGAGTA

oSNP_c841_2R AGCCTGACAGGTCGCATAAA G/T

oSNP_c841_3F TGTCAATTTCTTGGGTTGGA

oSNP_c841_3R CTCACGGCCAATTAAACTACG G/T

oSNP_c876_1F TCATGCTCAGTGGTGAAAGC

oSNP_c876_1R ACGCAGATACGAGCCATGAT G/T

oSNP_c1196_1F AGCCAATCAAGGTTGCATCT

oSNP_c1196_1R CAAACAGGGCGAACAGAGTT A/T

oSNP_c1361_1F TTGAGCATGAAGAAGACTCTGTG

oSNP_c1361_1R TCACATGGTTTAGCACATGAAA A/C

oSNP_c1579_1F GTCTTCACCTCCGACATGG

oSNP_c1579_1R AGAAGGCGTCCTTGCTGTC G/C

oSNP_c2043_1F AACCGAGCTCAAGGACCTCT

oSNP_c2043_1R ATAACCACGGCCTTCCTGTT G/C

oSNP_c2106_1F GATCGCGATTTAATTTTAACTTTTT

oSNP_c2106_1R TCGTGTCCGGTTATGGGATA ?

oSNP_c2106_2F GGGTTGAGGAAGCTCAAGG

oSNP_c2106_2R ACAAGTTCGGAGCCTTTGTG ?

oSNP_c2391_1F GCTGCGCATCAAAGTTCTCT

oSNP_c2391_1R CCTCTAGCGCCTTCCTGTAG T/C

oSNP_c2391_2F CTACAGGAAGGCGCTAGAGG

oSNP_c2391_2R TCTTAAAGCTGGGGATGACG A/C

oSNP_c2391_4F CTGCAGCAATCATCGAGTTC

oSNP_c2391_4R CAGCTGGCATATCAAGCAGA G/C

oSNP_c2391_5F GCACAATCCAAGCTAACAAGG

oSNP_c2391_5R TCAAATCAGCGGATCATCAA G/T

oSNP_c2539_1F TATGCTGCTGCATTGTCCAC

oSNP_c2539_1R TGTGACATTGTTGCGTTCAA G/A

oSNP_c2680_1F CGGTCTAGGGATGTCTGGAA

oSNP_c2680_1R TAGTTCGCCAAGCTCTCGAT T/C

oSNP_c2760_1F AAGGGCGTGTACGTCTCTGT

oSNP_c2760_1R GCTCAATGTGGGAGACACC G/T

oSNP_c3212_1F GCCTGTTCCAGTGATTGTCC

oSNP_c3212_1R GCCGCTGCAACAGTAGAAGT G/A

oSNP_c3768_1F AAGGCCGTGGTCTACTGGAT

oSNP_c3768_1R GTGGCGATCTTGAGGAGGAG G/A

oSNP_c4096_1F TAGCAAGAGCACTGGAAACG

oSNP_c4096_1R GACAGCATCTAGCCCTGGAG T/C

oSNP_c5153_1F CGTTGCTGGTGTTGAAGCTA

oSNP_c5153_1R TTTTCGGCTTCCTTATTGGA G/A

oSNP_c5252_1F TTCAATTGAGAAGGGCATCG

oSNP_c5252_1R ACCCCTCAACTAGACCTGGA G/A

oSNP_c5469_1F CCTTGACTCTGGGAACAAGC

oSNP_c5469_1R TGAACTGGAAGTGCATGAGC G/A

oSNP_c7461_1F TCGCTATAACGCGCAAAAA

oSNP_c7461_1R GGAGCTGATTGGAAGGTTGA G/A

oSNP_c10486_1F AGATTCTGGCTCCGATCAAA

oSNP_c10486_1R GGACACAGGTTCTCTCCACAA G/T

oSNP_c11164_1F AGGAGCCCCACCCCTAAT

oSNP_c11164_1R CTGCTCTCCCTTGGTACAGG G/A

oSNP_c11164_2F CCCGGTAGACCATAATATCTGC

oSNP_c11164_2R TGAGCACAAGATTATTTGATGGA T/C

oSNP_c12344_1F TCCGCCTAGTAACCACCAAC

oSNP_c12344_1R TTGCCAATGTCACCTGCATA G/C

oSNP_c12516_1F TCTCATGGATGATGGTGTGG

oSNP_c12516_1R GGGGTCCATCAAGATTCAGA A/T

oSNP_c12516_2F ATGTCGTTGCTACCGTGTCT

oSNP_c12516_2R TGCACACATTGAAACATACTGG G/A

oSNP_c14852_2F TTGGGTGTAAATTTCCTGTGC

oSNP_c14852_2R GGCCACTCACTCACCTACTCA A/C

oSNP_c15098_1F ACCGCCTAACATCAATTCCA

oSNP_c15098_1R AAAAGAAAACCATTGTCGATCC T/C

oSNP_c16908_1F CTTCCTGGTCTTCTCCGTGT

oSNP_c16908_1R TCACTATTTGCCGATGTGGA G/A

oSNP_c22314_1F TTTTTCAAATTCGACAACAAAG

oSNP_c22314_1R CAGAAATCCATCCAAGTTCCA G/T

oSNP_c23257_1F GATGGCACGTGGTGTTATTG

oSNP_c23257_1R GCGGTAGAATGCCTCGATA A/C

oSNP_lrc14030_1F ATGGCCTCAACGAGATCAAG

oSNP_lrc14030_1R TGACAACGATAGAACAGACCAAA A/T

oSNP_lrc16053_1F GCCAGACATCTTCAGCGTAA

oSNP_lrc16053_1R GCCATGTACCCAAACAGGAA G/A

oSNP_lrc16053_2F TAGTCTGCCCAAGGATGGAG

oSNP_lrc16053_2R GTCAACATGCCCCAGGAC T/C

oSNP_lrc16053_3F GGGCATGTTGACAGACTCCT

oSNP_lrc16053_3R ACGTTCTCCCCTCAGTGGTT T/C

oSNP_lrc27472_2F ATGCAATTCCCCACCATGT

oSNP_lrc27472_2R GAGGTGGGGGTGGTTGTG A/C

oSNP_lrc34490_1F TAAGGGAAAGTCACGCCTTG

oSNP_lrc34490_1R CAGTGGTCAGGCAAAGAACA A/C

oSNP_lrc38531_1F ACGATCACGAAAAGGGTCAC

oSNP_lrc38531_1R TCCTGTTGTCTGCAGTTTGTG A/C

oSNP_lrc38531_2F GCAATGTCATCTTCGGATTC

oSNP_lrc38531_2R TCTGGCTGGCTGTCTAGTGA ?

oSNP_lrc40347_1F CCTGTACTTGGTGGCGATGT

oSNP_lrc40347_1R TACGGCCAAATTAACCAAGC G/C
